# Early tapering of immunosuppressive agents after HLA-matched donor transplantation can improve the survival of patients with advanced acute myeloid leukemia

**DOI:** 10.1007/s00277-017-3204-6

**Published:** 2017-12-18

**Authors:** Jun Yang, Yu Cai, JieLing Jiang, LiPing Wan, HaiTao Bai, Jun Zhu, Su li, Chun Wang, Xianmin Song

**Affiliations:** Department of Hematology, Shanghai General Hospital affiliated to Shanghai Jiao Tong University, Haining road 100, Shanghai, 200080 China

**Keywords:** Acute myeloid leukemia (AML), Allogeneic, Hematopoietic stem cell transplantation (HSCT), Immunosuppressive agents

## Abstract

Disease recurrence is the most important obstacle to achieve long-term survival for patients with advanced acute myeloid leukemia (AML) after allogeneic hematopoietic stem cell transplantation (allo-HSCT). In order to reduce the relapse risk and improve the survival, the strategy of early tapering of immunosuppressive agents was prospectively evaluated. Thirty-one patients with advanced AML received early tapering of immunosuppressive drugs, while 32 patients with AML in complete remission (CR) were given the routine tapering of immunosuppressive agents after HLA-matched donor transplantation. All advanced AML patients achieved CR after allo-HSCT. At 24 months after transplantation, relapse incidences were 22% in advanced group and 16% in CR group (*P* = 0.553); disease-free survival (DFS) and overall survival (OS) were 57.7 and 57.8% in advanced group, while in CR group were 66.6% (*P* = 0.388) and 66.2% (*P* = 0.423); immunosuppressive agent-free DFS (IDFS) were similar between two groups (*P* = 0.407). Acute graft-versus-host disease (aGvHD) incidences were similar between two groups (*P* = 0.311). Chronic GvHD (cGvHD) incidence was much higher in advanced group than in CR group (70.4 vs 38.7%, *P* = 0.02), but severe cGvHD had no difference. In multivariate analysis, cGvHD was an independent prognostic factor for lower risk of relapse and better DFS and OS; early tapering of immunosuppressive agents was an independent prognostic factor for cGvHD. The study suggested that advanced AML patients could be directly treated with allo-HSCT and its survival could be improved through the strategy of early tapering of immunosuppressive agents without significant adverse effects (Clinicaltrials.org NCT03150134).

## Introduction

Patients with AML refractory to initial and re-induction have dismal prognoses if they do not undergo allogeneic hematopoietic stem cell transplantation (allo-HSCT). However, several retrospective studies have reported long-term survival rates only of 10 to 32% for patients with AML not in remission at the time of allo-HSCT [1–4]. Leukemia progression was the most important reason of transplantation failure (42% for AML) for those patients with advanced disease [5]. Thus, how to decrease the recurrence rate and improve the survival for patients with advanced AML after transplantation is still a huge challenge.

Increasing the preconditioning intensity and strengthening the graft-versus-leukemia (GvL) effects are the two most important strategies to prevent relapse of patients with advanced AML. While the benefit of reducing the relapse risk through the intensified conditioning is offset by increasing the acute graft-versus-host disease (aGvHD) and non-relapse mortality (NRM) with a survival from 23 to 42% [6–8]. Apart from the intensified conditioning regimens, curative potential of allo-HSCT is largely based on immune-mediated GvL effects caused by donor T cells in the graft. Strategies of immune modulation such as donor lymphocyte infusion (DLI) and withdrawal of immunosuppression drugs are proved to be able to enhance GvL effects and decrease the relapse risk. Mounting evidences demonstrated that prophylactic DLI (pDLI) and preemptive DLI for high-risk AML are effective to prevent the relapse, but the results are varied from each centers. Liga M et al. [9] reported that leukemia patients receiving low-dose pDLI after allo-HSCT is associated with a relatively high incidence of severe GvHD. To eliminate the onset of severe aGvHD, pDLI was always infused after + 100 days, but some patients in advanced stage might relapse early after transplantation without chance to receive pDLI. Furthermore, the deficiency of donor lymphocytes will limit access to this treatment and the DLI process itself is more complicated.

Withdrawal of immunosuppressive drugs is generally accepted as first-line treatment for relapsed patients after allo-HSCT. Early withdrawal of immunosuppression can prevent overt morphologic relapse and get a durable remission for 10% of patients with relapsed AML after transplantation [10, 11]. In a retrospective analysis, Sairafi et al. [12] demonstrated that early immune intervention in cases of impending relapse was more effective compared with late intervention after overt relapse. Since withdrawing immunosuppression allows for strengthening GvL effects, early tapering of immunosuppressive drugs may be the most feasible and effective means to prevent relapse of advanced AML after allo-HSCT. To evaluate the effects and adverse impacts of early tapering of immunosuppressive agents, a prospective clinical trial was designed and proceeded in our institute. This trial was registered at www.Clinicaltrials.org as NCT03150134.

## Patients and methods

### Patients and study designs

**Fig. 1:**
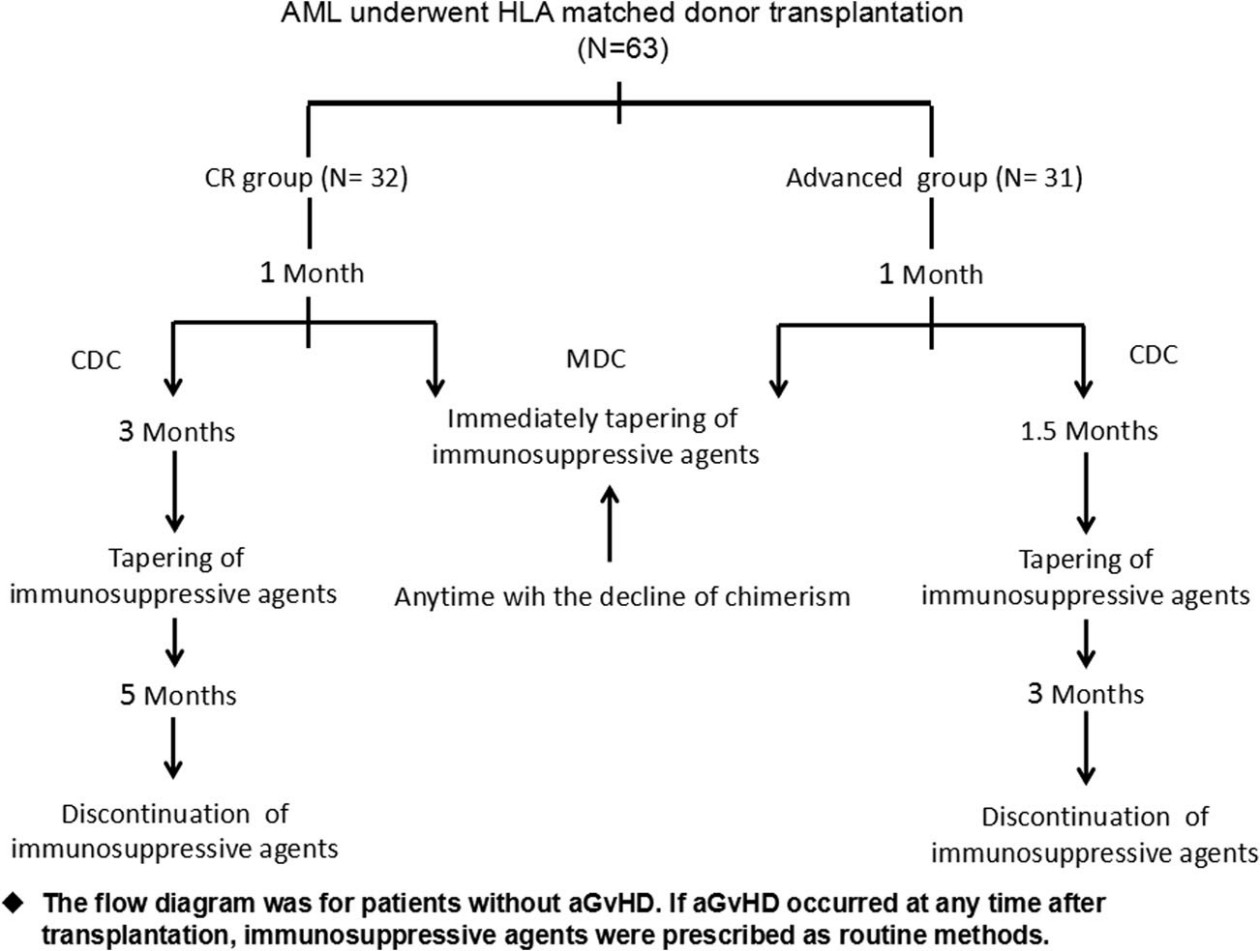
The flow diagram of immunosuppressive agent modulation after HLA-matched donor transplantation. *CDC*, complete donor chimerism; *MDC*, mixed donor chimerism; *GvHD*, graft-versus-host disease

Between January 1, 2010, and September 30, 2016, 63 consecutive patients with AML aged from 15 to 62 years were recruited in our clinical trial. The study was approved by the ethics committee of Shanghai General Hospital and conducted in accordance with the Declaration of Helsinki. Before enrollment, written informed consent was obtained from all patients or their legal guardians. All patients were followed up from the time of transplantation until the end of January 2017. The patients with AML were assigned into the different immune modulation groups after transplantation according to their disease status. Patients with advanced AML received early tapering of immunosuppressive drugs, while patients with AML in CR were given the routine tapering of immunosuppressive drugs (Fig. 1).

### Transplant procedures

Donor-recipient human leukocyte antigen (HLA) matching was considered for HLA-A, HLA-B, HLA-C, HLA-DR, and HLA-DQ. The HLA compatibility of donor-recipient pairs was defined as matched if they were at least 9/10 from sibling and unrelated donors. The grafts were all from mobilized peripheral blood with granulocyte colony-stimulating factor (G-CSF).

All the patients received myeloablative conditioning regimens (MCA). The conditioning regimen in this study consisted of total body irradiation (TBI) 3 Gy for all patients on day 1, intravenous busulfan 3.2 mg/kg/day from days 6 to 4, fludarabine 30 mg/m^2^/day and cytarabine (Ara-C) 1.5 g/m^2^/day from days 6 to 2 in 33 patients (BFAT), or cladribine (5 mg/m^2^/day × 5 days) substituted fludarabine in 30 patients from the year 2014 (BCAT). For two patients over 60 years, busulfan in BFAT regimen was reduced for 2 days. 2.5 mg/kg/day rabbit antithymocyte globulin (ATG) for two consecutive days (days 3 and 2) was administered for 34 patients receiving HLA-matched unrelated donor transplantation.

GvHD prophylaxis was cyclosporine (CsA) in combination with short-term methotrexate. CsA was dosed intravenously starting on day 7 to achieve a target trough level of 200–300 ng/mL. Intravenous methotrexate was delivered on days + 1, + 3, and + 6 after graft infusion.

All patients received prophylactic levofloxacin, acyclovir from the beginning of conditioning therapy until hematological reconstitution. Prophylactic fluconazole or posaconzale was administered from the day of the conditioning until 1 month after transplant [13]. Quantitative real-time PCR assays for cytomegalovirus (CMV) DNA in serum were also performed and preemptive therapy with ganciclovir (5 mg/kg, twice) started if CMV DNA was more than 1000 copies/mL.

### Chimerism monitoring

Quantitative chimerism monitoring [14] was performed by short-tandem repeat-based PCR techniques on CD3-positive cell population from bone marrow and by fluorescent in situ hybridization (FISH) [15] for patients with sex-mismatched donors at regular intervals for every 4 weeks after transplant at first 6 months. Mixed T cell chimerism was defined as between 5 and 94% recipient cells and complete donor chimerism (CDC) was defined as the presence of more than 95% donor chimerism at all measured time points [16].

### Immunosuppressive agent modulation post transplantation

Immunosuppressive agents were adjusted according to the schedule (Fig. 1). Usually, in the absence of aGvHD, immunosuppressive agents were gradually reduced by 6 weeks and discontinued at 3 months after transplant for patients with advanced AML even if CDC was achieved. If donor chimerism did not achieve CDC with no significant aGVHD at 4 weeks after HSCT, immunosuppressive agents were gradually reduced. If aGvHD was present during tapering of immunosuppressive agents, CsA was added again and tapering was done over a longer period. Immunosuppressive agents were regularly tapered by 3 months and discontinued at 5 months after transplant for AML patients in CR without aGvHD. CsA was withdrawn by 25% per week for patients with active disease and by 10% per week for patients with CR without aGVHD by schedule time according to the flow diagram.

### Statistical methods

We calculated DFS in patients with CR from the time of achieving remission after transplantation to the time of relapse, death from any cause, or last follow-up. For DFS, an event was defined as relapse or death from any cause. For immunosuppressive agent-free DFS (IDFS), events included discontinuation of immunosuppressive drugs, relapse, or death from any cause. OS was defined from the date of allo-HSCT until the date of event occurrence or censored at last follow-up for patients without an observed event death from any cause. Alive patients were censored on the date of their last follow-up.

Kaplan-Meier estimates of DFS, OS, and IDFS were compared between groups via log-rank statistics and the Cox proportional hazards model [17]. The cumulative incidence of relapse (CIR) was calculated from the date of allo-HSCT or the date of getting CR after transplantation until relapse. The response criteria for CR and relapse were defined according to the literature [18]. Primary refractory disease was defined as the failure of achieving CR after two cycles of initial induction chemotherapy or hematologic relapse within 6 months from the beginning of initial therapy. Secondary refractory disease was defined as relapse from CR and had no response to salvage chemotherapy. NRM was defined as death without evidence of disease relapse. The patients with advanced diseases who had no documented post HSCT CR were excluded from the final evaluation. aGvHD was diagnosed and graded according to the modified Glucksberg grading of aGVHD [19]. cGvHD was diagnosed and graded according to the 2014 National Institutes of Health (NIH) consensus criteria: mild, moderate, or severe, respectively [20]. Neutrophil engraftment was defined as the first of three consecutive days of count > 0.5 × 10^9^/L. Platelet engraftment was defined as the first of seven consecutive days with platelet counts of > 20 × 10^9^/L without platelet transfusion.

Chi-squared and independent sample *t* test were used to compare the frequency distributions of variables such as age, sex, risk factor at diagnosis, median time from diagnosis to HSCT, donor characteristic, acute, and cGvHD rates. Multivariate analyses for differences between two groups were performed using Cox proportional hazards regression model. Multivariate analyses for risk factors related to cGvHD were performed by the sequential logistic regression model, respectively. All statistical tests were two-sided and *P* value < 0.05 was considered significant. The statistical analyzes were performed using IBM SPSS 17.0 statistical software (IBM, North Harbour, Portsmouth, UK).

## Results

### Patient characteristics

The patients’ characteristics were summarized in Table 1. Sixty-three patients with AML (median age 38 years, range 15–62) were diagnosed and recruited during the study period. One patient was secondary to MDS-RAEB II without any response to decitabine-based therapy. Three patients were diagnosed as secondary AML following radiotherapy or chemotherapy for gastrointestinal tumor, breast cancer, or uterine cancer. The other 59 patients were diagnosed as de novo AML. At the time of transplantation, a total of 32 patients reached first or subsequent complete response (CR1, CR2) with conventional therapy or salvage therapy; the other 31 patients had advanced disease without responding to salvage therapy and the median BM blasts at transplantation were 33% (range 10–78%). There was no difference between the two groups except that more patients in advanced group had high-risk factors at diagnosis (*P* = 0.001).

**Table 1:** Patient characteristics

	CR group	Advanced group	*P* value
No. patients	32	31	
Median age, years (range)	37 (15–52)	41 (19–62)	*P* = 0.24
Sex			*P* = 0.32
Male	18	13	
Female	14	18	
Diagnosis			*P* = 0.479
De novo AML	31	28	
Secondary AML	1	2	
MDS-AML	0	1	
Risk classification at diagnosis*			*P* = 0.001
High	11	24	
Intermediate	21	7	
Median interval from diagnosis to HSCT, months (range)	8 (3–19)	8 (3–68)	*P* = 0.096
Tapering of immunosuppressive agents			
Early	7	22	*P* < 0.001
Regular	25	9	
Disease status at transplantation			
CR1	25		
CR2	7		
Secondary refractory		23	
Primary refractory		8	
Donor characteristics			*P* = 0.556
Matched related (10/10)	15	14	
Matched unrelated (10/10)	10	12	
Matched unrelated (9/10)	7	5	
Conditioning regimens			*P* = 0.359
BFAT	18	15	
BCAT	14	16	

*CR* complete remission, *AML* acute myeloid leukemia, *MDS-AML* myelodysplastic syndrome-related acute myeloid leukemia, *BFAT* busulfan+fludarabine+Ara-C+TBI, *BCAT* busulfan+cladribine+Ara-C+TBI

*Risk classification at diagnosis was evaluated according to the cytogenetics at the time of diagnosis [21]

### Engraftment and GvHD

All patients were successfully engrafted. The median time for neutrophil engraftment was 12 days (range 8–15) and 13 days (range 10–16) in CR and advanced recipients, respectively (*P* = 0.162), whereas the median time for platelet engraftment was observed in 12 days (range 8–15) and 13 days (10–31) in the two groups, respectively (*P* = 0.019).

The cumulative incidence (CI) within day 100 of grade II–IV aGvHD was 34.4% in CR group and 48.4% in advanced group (*P* = 0.311), while the CI of grade III–IV aGvHD was 9 and 9.7%, respectively. Among the evaluable 58 patients, the CI of cGvHD at 2 years was much higher in advanced group than in CR group (70.4 vs 38.7%, *P* = 0.02), but the CIs of severe cGvHD were similar between two groups (14.8% in advanced group and 9.7% in CR group, *P* = 0.694).

### Chimerism analysis and immunosuppressive agent modulation

At 4 weeks after transplantation, complete donor chimerism (CDC) was found in 58 patients, while 5 patients had a mixed chimerism (3 in advanced group and 2 in CR group). Immunosuppressive agents were gradually reduced in these five patients. Three of them achieved CDC after 2–4 weeks and they all had a long survival. Two patients in advanced group soon died with relapse at 2 months after transplantation. Early tapering of immunosuppressive agents was performed for another five patients in CR group because of the decline of the chimerism at 6 weeks or 2 months after transplant. One patient died from aGvHD at 10 weeks and the other four patients had a long survival without relapse. Donor chimerism was decreased in seven patients at the time of hematologic relapse and two patients still had the CDC at the time of isolated extramedullary relapse. Immunosuppressive agents were regularly reduced in nine patients who were in advanced group because of aGvHD (grades II–III). Immunosuppressive agents were modulated as study design for all other patients.

### Disease response and survival

**Fig. 2:**
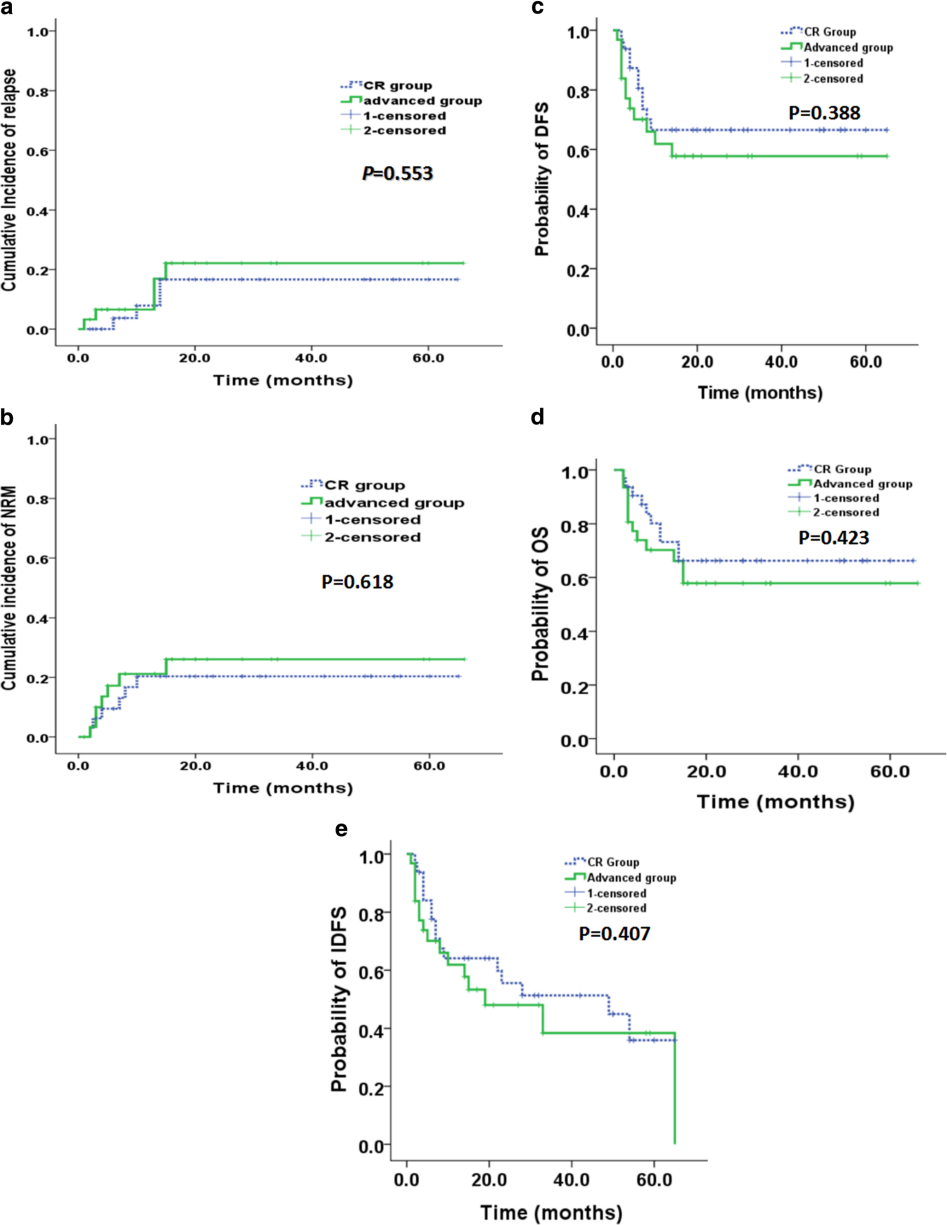
Clinical outcomes of patients in CR group and advanced group with allo-HSCT. **a** Cumulative incidence (CI) of relapse, **b** non-relapse mortality (NRM**)**, **c** disease-free survival (DFS**)**, **d** overall survival (OS), and **e** immunosuppressive agent-free DFS (IDFS)

The median follow-up time for these patients was 15 months (range 2–66 months). All the patients with advanced disease before transplant achieved CR after allo-HSCT. Four cases in CR group and five cases in advanced group died due to relapse in the first year after allo-HSCT. All the nine patients had no significant GvHD even after the reduction of immunosuppressive agents. The median time of relapse was 5.5 (2–10) months and these patients died at 1–8 months after disease relapse. At 2 years, CIs of relapse were similar in two groups (16% in CR group vs 22% in advanced group, *P* = 0.553) (Fig. 2a). Six patients died from pneumonia (3 in CR group and 3 in advanced group), while seven patients died from aGvHD (3 in CR group and 4 in advanced group). Totally, ten patients in CR group died while 12 patients in advanced group. At 2 years, CIs of NRM were similar in two groups (20% in CR vs 26.1% in advanced group, *P* = 0.618) (Fig. 2b). No significant differences between CR and advanced groups were observed at 2-year DFS (66.6% [95% CI, 49.55–83.65%] vs 57.7% [95% CI, 39.08–76.32%], *P* = 0.388) (Fig. 2c) and OS (66.2% [95% CI, 49.15–83.25%] vs 57.8% [95% CI, 39.18–76.42%], *P* = 0.423) (Fig. 2d) after transplantation.

At the end of follow-up, six patients in CR group and four patients in advanced group still had cGvHD with immunosuppressive treatment. All these patients had a long survival (median survival 25.5 months, range 4–66). No significant differences were observed at 2-year immunosuppressive agent-free DFS (IDFS) (51.3% [95% CI, 32.5–70.11%] in CR group vs 48% [95% CI, 28.2–67.8%], *P* = 0.407) (Fig. 2e) in advanced group after transplantation.

### CMV infection

Seventeen patients (53.1%) in CR group and 18 (58.06%) patients in advanced group were CMV seropositive (*P* = 0.801) after transplantation. The median time of CMV seropositive was 4 (3–6) months in CR group and 4 (3–8) months in advanced group, respectively. All of them were successfully treated with antiviral strategies.

### Univariate and multivariate analyses for relapse risk, DFS, and OS after HSCT

**Fig. 3:**
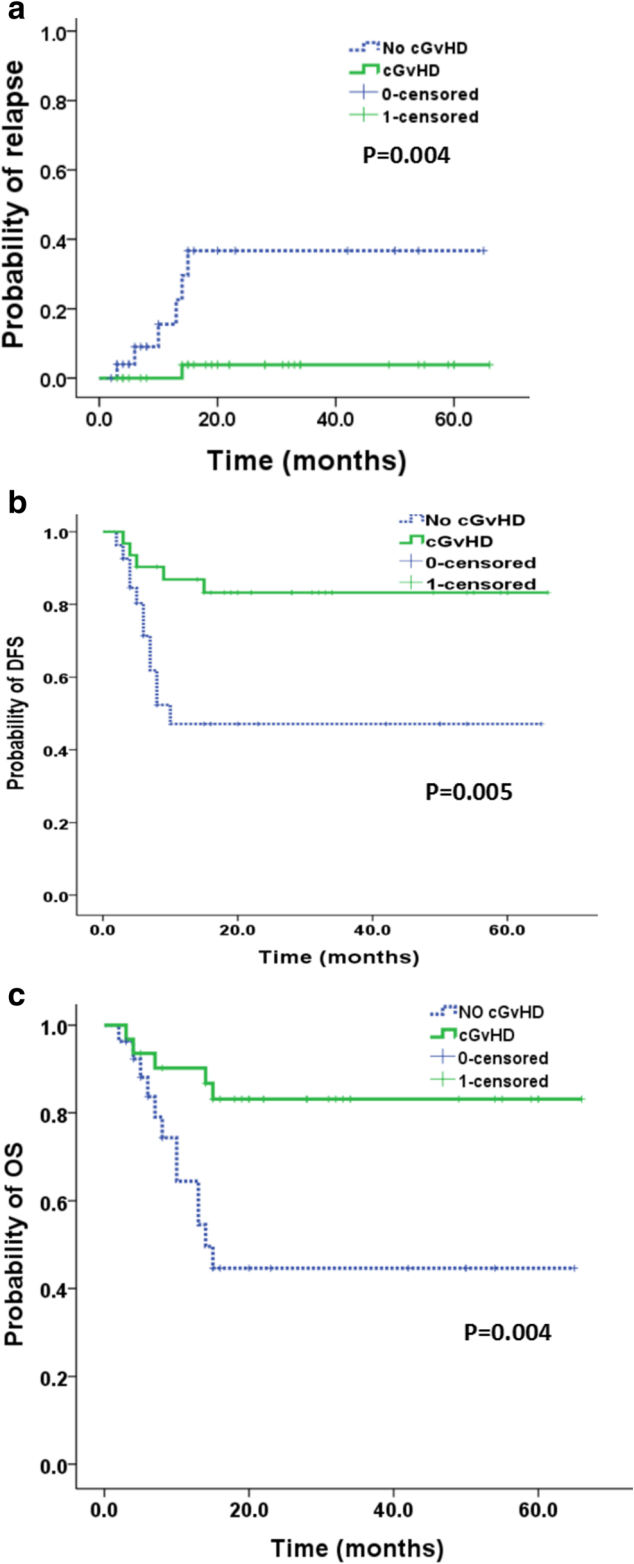
Clinical outcomes of patients with and without chronic graft-versus-host disease (cGvHD). a Cumulative incidence (CI) of relapse; b disease-free survival (DFS), and c overall survival (OS)

Univariate and multivariate analyses for relapse, DFS, and OS were shown in Tables 2 and 3, respectively. Using Cox proportional hazards regression models, two factors were demonstrated to have a significant impact toward a lower HR of relapse in univariate analysis, including (1) cGvHD: HR 11.838 [95% CI, 1.416–98.95], *P* = 0.023 and (2) CMV viremia: HR 11.905 [95% CI, 1.483–95.55], *P* = 0.02. Only one positive prognostic factor of cGvHD was associated with DFS (HR 4.067 [95% CI, 1.421–11.641], *P* = 0.009) and OS (HR 4.145 [95% CI, 1.452–11.838], *P* = 0.008). Factors related to relapse risk, DFS, and OS with *P* value less than 0.4 in univariate analysis were subsequently included in the multivariate analysis. The results revealed that cGvHD and CMV viremia were the independent prognostic factors with a lower relapse risk, while only cGvHD as one independent prognostic factor was associated with a better DFS and OS (Table 3) (Fig. 3).

**Table 2:** Univariate analysis for relapse risk, DFS, and OS

Characteristics	Relapse		DFS		OS	
	HR (95% CI)	*P*	HR (95% CI)	*P*	HR (95% CI)	*P*
Age (15–40 vs > 40years)	0.572 (0.143–2.295)	0.431	1.271 (0.548–2.949)	0.576	1.308 (0.564–3.032)	0.532
Gender (male vs female)	0.798 (0.214–2.974)	0.737	0.816 (0.352–1.889)	0.634	0.831 (0.359–1.924)	0.665
Risk classification at diagnosis (intermediate risk vs high risk)	0.596 (0.149–2.383)	0.464	0.996 (0.43–2.305)	0.992	0.993 (0.429–2.3)	0.988
HLA matched (10/10 matched vs 9/10 matched)	2.547 (0.318–20.375)	0.378	1.099 (0.405–2.979)	0.853	1.077 (0.397–2.921)	0.884
Median interval from diagnosis to HSCT (< 12 vs ≥ 12 months)	0.637 (0.159–2.549)	0.524	0.866 (0.339–2.215)	0.764	0.855 (0.334–2.186)	0.743
BM blasts at transplantation (< 20 vs ≥ 20%)	1.163 (0.242–5.602)	0.85	1.065 (0.393–2.889)	0.901	1.108 (0.409–3.005)	0.84
Conditioning regimen (BFCT vs BCAT)	0.411 (0.103–1.645)	0.209	0.622 (0.265–1.455)	0.273	0.59 (0.252–1.381)	0.224
aGvHD of grades II–IV (with vs without)	5.185 (0.648–41.495)	0.121	0.701 (0.304–1.617)	0.404	0.664 (0.288–1.533)	0.338
cGvHD (with vs without)	11.838 (1.416–98.95)	0.023	4.067 (1.421–11.641)	0.009	4.145 (1.452–11.838)	0.008
Severe cGvHD (with vs without)	0.551 (0.114–2.66)	0.458	1.169 (0.267–5.114)	0.836	1.183 (0.27–5.177)	0.823
CMV viremia (with vs without)	11.905 (1.483–95.55)	0.02	1.452 (0.628–3.359)	0.383	1.401 (0.607–3.246)	0.428

**Table 3:** Multivariate analysis for relapse risk, DFS, and OS

	Relapse		DFS		OS	
	HR (95% CI)	*P*	HR (95% CI)	*P*	HR (95% CI)	*P*
cGvHD (with vs without)	35.02 (2.104–582.791)	0.013	5.074(1.666–15.455)	0.004	5.603(1.802–17.421)	0.003
CMV viremia (with vs without)	46.321 (1.717–1249.9)	0.023

### Univariate and multivariate analysis for cGvHD

We also analyzed the prognostic factors related to cGvHD using univariate and multivariate analyses with the sequential logistic regression model. The results were shown in Tables 4 and 5, respectively. In univariate analysis, early tapering of immunosuppressive therapy (*P* = 0.004), risk classification at diagnosis (*P* = 0.079), BM blasts at transplantation (*P* = 0.053), and aGvHD (*P* = 0.047) were the risk factors associated with cGvHD (Table 4).

**Table 4:** Univariate analysis for cGvHD

Characteristics	HR (95% CI)	*P* value
Age(15–40 vs > 40years)	0.486 (0.169–1.395)	0.180
Gender (male vs female)	0.57 (0.2–1.623)	0.293
Risk classification at diagnosis (intermediate risk vs high risk)	0.384 (0.132–1.116)	0.079
HLA matched (10/10 matched vs 9/10 matched)	1.316 (0.394–4.393)	0.655
Median interval from diagnosis to HSCT (< 12 vs ≥ 12 months)	0.608 (0.176–2.109)	0.433
BM blasts at transplantation (< 20 vs ≥ 20%)	0.249 (0.061–1.017)	0.053
Initiate time of tapering of immunosuppressive agents (early vs routine)	5.182 (1.669–16.085)	0.004
Conditioning regimen (BFCT vs BCAT)	0.971 (0.344–2.742)	0.956
aGvHD of grades II–IV (with vs without)	3.076 (1.013–9.338)	0.047
CMV viremia (without vs with)	0.684 (0.241–1.942)	0.476

**Table 5:** Multivariate analysis for cGvHD

	cGvHD	
	HR (95% CI)	*P* value
Initiate time of tapering of immunosuppressive agents (early vs routine)	4.971 (1.34–18.442)	0.017
aGvHD (with vs without)	4.015 (1.11–14.531)	0.034

Factors related to cGvHD with *P* value less than 0.1 in univariate analysis were subsequently included in the multivariate analysis (Table 5). The results showed that early tapering of immunosuppressive drugs might increase the incidence of cGvHD (HR 4.971 [95% CI, 1.34–18.442], *P* = 0.017) and aGvHD also has positive relationship with the incidence of cGvHD (HR 4.015 [95% CI, 1.11–14.531], *P* = 0.034).

## Discussion

In this pilot study, the results showed that the transplant outcomes of patients with AML in advanced stage were similar with patients in CR. The DFS and OS of patients in advanced stage were 57.7 and 57.8%, respectively, which were much better than that from previous reports with long-term survival only of 10 to 32% for patients with AML not in remission at the time of allo-HSCT [1–4]. Duval et al. [5] reported a large series of 1673 adult and pediatric patients with refractory AML in non-remission only achieved a 3-year OS of 19%, whereas with a high 3-year TRM of 39% after allo-HSCT with a myeloablative procedure. Their results also indicated that lower-risk patients with active AML could benefit from the transplantation, while high-risk patients could not benefit from immediate transplantation and should achieved CR before transplantation [5]. A recently retrospective study [22] compared the transplant results of patients in different stages like CR2 (*n* = 1986), primary induction failure (PIF) (*N* = 1440), and first relapse (Rel1) (*N* = 1256). The 5-year survival adjusted for performance score, cytogenetic risk, and donor type for CR2 patients was 39% (95% confidence interval [CI], 37–41%) compared with 18% (95% CI, 16–20%) in Rel1 and 21% (95% CI, 19–23%) in PIF (*P* < .0001) for allo-HSCT. This study had similar conclusion with Duval’s report about the important value of disease remission when transplantation. As compared with these retrospective analyses, our study had a completely different result that the survival of patients with active AML after allo-HSCT was similar with the survival of patients in CR. That implied that one patient with advanced AML could be cured through immediate allogeneic transplantation and the strategy of early immunosuppressive drug modulation.

The results of this study suggested that the GvL effect was vitally important for patients with advanced AML to achieving a long-term survival. The GvL effects commonly accompanied with cGvHD have been demonstrated in many studies. The higher incidence of cGvHD (74%) in advanced group might be the pivotal reason in our study for a similar survival with patients in CR. In accordance with other reports [6, 23–27], our results showed that cGvHD was associated with a lower risk of relapse and better OS and DFS. Severe cGvHD is usually associated with higher TRM. However, in the present study, the incidences of severe cGvHD in advanced group (14.8%) were similar to those in CR group (9.7%) (*P* = 0.694). And ultimately, there was no significant difference in the incidences of TRM between two groups (20 vs 26.1%, *P* = 0.618). At the end of follow-up, many patients in advanced group had been cured without any immunosuppressive agents for cGVHD. The 2-year IDFSs were similar between two groups (51.3% in CR group vs 48% in advanced group, *P* = 0.407) (Fig. 2e) after transplantation. Four patients in advanced group needed continued immunosuppressive treatment because of cGvHD. But only one patient of them was diagnosed with severe cGvHD. The other three patients were suffered from oral ulcer which did not affect the quality of life.

In the present study, cGvHD was only associated with early tapering of immunosuppressive agents and aGvHD according to multivariate analysis. But the incidence of aGvHD is similar in two groups. Our strategy of early tapering of immunosuppressive agents resulted in a higher incidence of cGvHD, but the cGvHD-related lethality was acceptable. Clinically, a significant GvL effect is induced by cGvHD rather than aGvHD [28, 29]. The higher incidence of cGvHD in the advanced group was associated with a lower probability of relapse after transplant in the present study, probably corresponding to a potential GvL effects. Withdraw of immunosuppressive agents was the first-line treatment strategy for the early relapsed or molecular relapsed AML after allo-HSCT, which was demonstrated to be able to cure these patients from mounting evidences [30–32]. That suggested that our strategy of immunosuppressive agent modulation to prevent relapse was effective and safe.

Leukemia burden at the time of transplantation is the most important prognostic factor except for other factors like cytogenetics, primary induction failure, and matched unrelated donor. Some studies showed that bone marrow blast cell count less than 20% was associated with improved long-term survival [33]. However, in our study, BM blasts at the time of transplantation has no significant relationship with DFS and OS. Early tapering of immunosuppressive agents could achieve GvL effects at early time after transplantation to prevent the relapse, which is one reason we speculated for no relationship between leukemia burden and survival, because the relapse most occurred at the first 6 months after transplantation. The other reason might be due to the low number patients. That needed to be further evaluated in a large number samples. All the patients with advanced AML achieved CR after transplantation, which suggested that the conditioning regimen is highly effective. The dose of busulfan in our conditioning was reduced to 3 days as compared with standard conditioning dose to lessen the toxicity of conditioning chemotherapy and decrease the NRM. The adding of cladribine to the standard induction regimen can improve the survival of the high-risk AML [34–36], so from 2014, cladribine was also used as one part of the conditioning regimens to substitute fludarabine in this study. But the multivariate analysis found that the conditioning regimen also had no significant relationship with DFS and OS.

Recent studies have reported a beneficial effect of early CMV reactivation after transplant to relapse prevention, but this effect seems unclear with regard to overall survival. This protective effect appeared to be particularly in AML patients [37–40]. Our finding was quite in accordance with these studies [41, 42]. In this study, our results also showed that CMV reactivation was the independent prognostic factors for a lower relapse risk but had no relationship with OS and DFS. Further research is needed.

In conclusion, our data suggested that the patients with advanced AML could be cured through immediate allo-HSCT. The strategy of early tapering of immunosuppressive agents could be effectively and safely performed to prevent the disease recurrence and improve the survival of AML patients after allo-HSCT.
